# B-cell receptor dependent phagocytosis and presentation of particulate antigen by chronic lymphocytic leukemia cells

**DOI:** 10.37349/etat.2022.00070

**Published:** 2022-02-25

**Authors:** Annabel R. Minton, Lindsay D. Smith, Dean J. Bryant, Jonathan C. Strefford, Francesco Forconi, Freda K. Stevenson, David A. Tumbarello, Edd James, Geir Åge Løset, Ludvig A. Munthe, Andrew J. Steele, Graham Packham

**Affiliations:** 1 Cancer Research UK Centre, Cancer Sciences, Faculty of Medicine, University of Southampton, SO16 6YD Southampton, UK; 2 Current address: Ploughshare Innovations Limited, Porton Science Park, Porton Down, SP4 0BF Wiltshire, UK; 3 Biological Sciences, Faculty of Environmental and Life Sciences, University of Southampton, SO17 1BJ Southampton, UK; 4 Nextera AS, Gaustadalléen 21, NO-0349 Oslo, Norway; 5 KG Jebsen Centre for B cell Malignancies, Institute of Clinical Medicine, University of Oslo, NO-0424 Oslo, Norway; 6 Current address: Janssen R&D, 1400 McKean Road, Spring House, Ambler, PA 19477, USA; Istituto Nazionale Tumori-IRCCS-Fondazione G. Pascale, Italy

**Keywords:** Chronic lymphocytic leukemia, antigen presentation, T helper cell, B-cell receptor, phagocytosis, major histocompatibility complex class II, human leukocyte antigen class II

## Abstract

**Aim:**

T-helper cells could play an important role in the pathogenesis of chronic lymphocytic leukemia (CLL), a common B-cell neoplasm. Although CLL cells can present soluble antigens targeted from the B-cell receptor to T-helper cells via major histocompatibility complex (MHC) class II, antigens recognized by some CLL cells may be encountered in a particulate form. Here the ability of CLL cells to internalize and present anti-immunoglobulin M (IgM) beads as a model for the interaction of CLL cells with particulate antigens was investigated.

**Methods:**

The effect of anti-IgM beads on antigen presentation pathways was analyzed using RNA-seq and internalization of anti-IgM beads by primary CLL cells was investigated using confocal microscopy and flow cytometry. Antigen presentation was investigated by analyzing activation of a T-cell line expressing a T-cell receptor specific for a peptide derived from mouse κ light chains after incubating CLL cells with a mouse κ light chain-containing anti-IgM monoclonal antibody. Kinase inhibitors were used to characterize the pathways mediating internalization and antigen presentation.

**Results:**

Stimulation of surface IgM of CLL cells increased expression of the antigen presentation machinery and CLL cells were able to phagocytose anti-IgM beads. Internalization of anti-IgM beads was associated with MHC class II-restricted activation of cognate T-helper cells. Antigen presentation by CLL cells was dependent on activity of spleen tyrosine kinase (SYK) and phosphatidylinositol 3-kinase delta (PI3Kδ) but was unaffected by inhibitors of Bruton’s tyrosine kinase (BTK).

**Conclusions:**

CLL cells can internalize and present antigen from anti-IgM beads. This capacity of CLL cells may be particularly important for recruitment of T-cell help *in vivo* in response to particulate antigens.

## Introduction

The ability of the B-cell receptor (BCR) to internalize antigen (Ag) for major histocompatibility complex (MHC) class II presentation to CD4^+^ T helper (Th) cells is critical for generating effective antibody responses [1, 2]. The mechanism by which the BCR internalizes Ag depends on the form of Ag [3–5] and although B cells were generally considered to have low phagocytic activity, it is now recognized that B cells can phagocytose and present particulate Ag, especially when targeted to the BCR [4, 6, 7]. The form of Ag recognized by the BCR has important consequences for shaping immune responses, and particulate Ag elicits particularly potent responses [8, 9].

BCR-induced signaling plays a key role in chronic lymphocytic leukemia (CLL), a common mature B-cell malignancy, and is an established target for therapy as illustrated by the use of the Bruton’s tyrosine kinase (BTK) inhibitor ibrutinib [10]. By contrast, the capacity of the BCR to internalize Ag for presentation by CLL cells to Th cells is much less well understood. However, several lines of evidence indicate an important role for Th cells in CLL pathogenesis [11]. Th cells that are capable of providing help to cognate CLL cells are present in the blood of CLL patients [12] and Th cells are juxtaposed with CLL cells within proliferation centers, sites of CLL cell proliferation in the lymph nodes and bone marrow of patients [13, 14]. Th cells are required for growth of CLL cells in xenograft models [15] and factors expressed by Th cells [such as interleukin-4 (IL-4) and CD40L] promote survival and/or proliferation of CLL cells *in vitro* [16–18].

CLL BCRs have been shown to bind a range of Ag and autoantigens [19]. Importantly, some of these candidate Ag, such as those from bacteria, viruses, fungi or apoptotic bodies/cell fragments [20–24], may be encountered *in vivo* in a particulate form. Whereas CLL cells have been shown to be able to present soluble Ag to Th cells following internalization via surface immunoglobulin M (sIgM) [12], it is not known whether CLL cells are also capable of internalizing and presenting particulate Ag. Here we have investigated the interaction of CLL cells with anti-immunoglobulin M (IgM)-coated beads as a mimic for particulate Ag.

## Materials and methods

### Compounds and antibodies for stimulation

Ibrutinib, acalabrutinib, entospletinib, idelalisib and dyngo-4A (SelleckChem), and cyctochalasin D (Merck) were dissolved in dimethyl sulfoxide (DMSO). The antibodies used were goat F(ab’)_2_ anti-human IgM, control goat F(ab’)_2_ and mouse F(ab’)_2_ κ anti-IgM (all Cambridge Biosciences). These were used as soluble antibodies or after covalent binding to 2.8 μm epoxy-coated M-280 Dynabeads (ThermoFisher) or 3 μm carboxylate-coated latex beads (Polybead^®^ Carboxylate Microspheres; PolySciences) using the manufacturers’ instructions.

### CLL samples

Analysis of CLL samples (Table S1) was performed following informed consent and in accordance with Ethics Committee approvals and the Declaration of Helsinki. Heparinized peripheral blood mononuclear cells (PBMCs) were obtained from patients attending clinic at the Southampton General Hospital. Immunoglobulin heavy chain variable region gene (*IGHV*) usage/mutation status, expression of CD5, CD19 and sIgM, and intracellular calcium responses following stimulation with soluble goat F(ab’)_2_ anti-IgM, were determined as previously described [25]. Cryopreserved samples were recovered and rested for 1 h in complete RPMI-1640 (RPMI-1640 medium supplemented with 10% fetal bovine serum, 2 mmol/L *L*-glutamine and 1% penicillin/streptomycin) at 37°C before use. Cell viability was determined by trypan blue exclusion and was ≥ 90% in all cases. Human leukocyte antigen (HLA)-typing was performed using the Olerup SSP HLA-DRB1-*04 HLA Typing Kit (CareDx).

### RNA-seq and bioinformatics

CLL samples (*n* = 6) were incubated with goat F(ab’)_2_ anti-human IgM or control antibody coated Dynabeads at a 2:1 bead to cell ratio for 6 or 24 h. Total mRNA was isolated using the Reliaprep™ RNA extraction kit (Promega, UK) and RNA-seq was performed at the Oxford Genomics Centre (Oxford, UK) using Illumina TruSeq Library Prep kit V2 and Illumina Novaseq 6,000 sequencing platform. RNA-seq data files are available at ArrayExpress (https://www.ebi.ac.uk/arrayexpress/) under accession E-MTAB-11195. Raw RNA-seq data (fastq files) was aligned against the hg19 reference genome using BWA and initial data quality control was performed using FastQC [26]. Counts matrices were produced using HTseq-count [27] and exported for differential expression analysis in EdgeR [28]. Transcriptomic data was fitted to multifactor generalized linear models and tested for differential expression using quasi-likelihood *F*-tests. Multiple testing correction was performed using the Benjamini-Hochberg procedure. Pathway analysis was performed using ingenuity pathway analysis (IPA; Qiagen).

### Flow cytometry

CLL samples were incubated with goat F(ab’)_2_ anti-human IgM or control goat F(ab’)_2_ antibody coated Dynabeads for 24 h. Cells were then stained with an adenomatous polyposis coli (APC)-conjugated anti-HLA-DR antibody (Becton Dickinson; 347403) and analyzed by flow cytometry.

### Bead internalization assays

Cells were incubated with goat F(ab’)_2_ anti-human IgM-coated or control antibody-coated latex beads at a 2:1 bead to cell ratio for 3 h at 37 or 4°C. Cells were then placed on ice for 10 min to terminate internalization. For confocal analysis, 2 × 10^6^ cells were incubated in 1× CellMask™ Deep Red solution (ThermoFisher) as per manufacturer’s instructions for 10 min at 37°C, placed on ice and fixed for 15 min using 4% paraformaldehyde. Samples were then washed and incubated with AlexaFluor488-conjugated donkey anti-goat IgG antibody (20 μg/mL; ThermoFisher A-11055) for 30 min on ice. Cells were washed and cytospun (Thermo Shandon Cytocentrifuge, C4) onto glass slides, dried for 10 min at room temperature and mounted using Vectashield Hardset mountant with 4’,6-diamidino-2-phenylindole (DAPI: Vector Laboratories, H-1500). Images were collected on a Leica SP5 CLSM with a 100× (NA1.4) Plan-apochromatic objective using LAS-AF software (Version2, Leica) and processed using Leica Application Suite X (Leica Microsystems). For flow cytometry, 1 × 10^6^ cells were stained with the AlexaFluor488-conjugated donkey anti-goat IgG antibody (20 μg/mL) for 30 min, washed and then analyzed.

### T-cell receptor cloning and generation of the SKW3-T18 cell line

The T-cell receptor (TCR) from the primary human T-cell clone T18 [29] was cloned and retrovirally reconstructed in TCR-negative murine BW58 T cells for verification of the TCR construct, as described [30]. Following functional verification, the TCR was introduced into the TCR-negative human SKW3 T-cell line (a derivative of the T-acute lymphoblastic leukemia cell line KE-37 [31]) to generate SKW3-T18 cells, as described [32]. DR4 tetramer reagents were purchased from Benaroya Research Institute (Seattle, WA) and used according to the manufacturer’s instructions.

### Ag presentation assay

SKW3-T18 cells were cultured in complete RPMI-1640. Cell line identity was confirmed using short tandem repeat analysis (Powerplex 16 System, Promega, Southampton, UK) and absence of mycoplasma was confirmed using Mycoplasma PCR detection kit (Applied Biological Materials, Richmond, Canada). CLL cells were purified from PBMCs using a B-CLL cell isolation kit (Miltenyi Biotec) and incubated with soluble antibody or antibody-coated beads (2:1 bead:cell ratio) for 15 or 60 min, respectively. CLL cells were then co-cultured with SKW3-T18 cells at a final density of 100,000 SKW3-T18 and 400,000 CLL cells in a total volume of 0.2 mL. Supernatants were collected after 24 h and IL-2 quantified using an enzyme-linked immunosorbent assay (ELISA; R&D Systems) according to the manufacturer’s instructions. All assays were performed in duplicate.

### Statistics

Statistical comparisons were performed using GraphPad Prism software (v9.2.0; GraphPad Software, La Jolla, CA, USA).

## Results

### sIgM stimulation of CLL cells increases MHC class II expression

**Figure 1.**
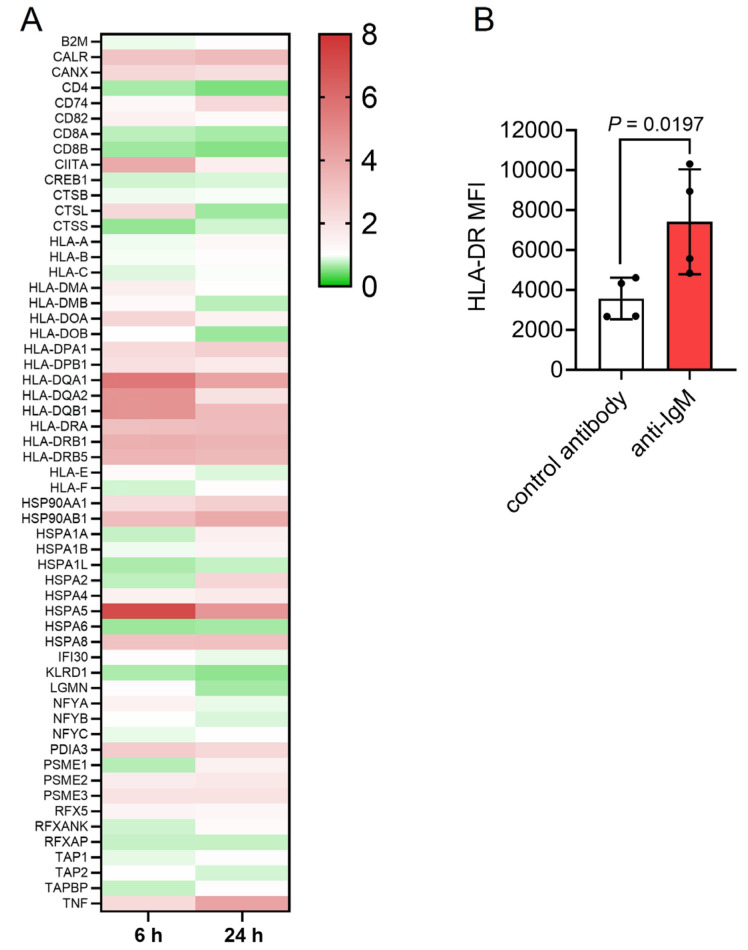
Effect of anti-IgM on Ag presentation-related genes. A: Heat-map showing fold changes in expression of detected genes induced following sIgM stimulation for 6 h or 24 h (with scale bar). Genes are from the KEGG Ag Processing and Presentation pathway (hsa04612); B: CLL samples (*n* = 4) were treated with Go anti-IgM or control antibody-coated Dynabeads for 24 h and expression of cell surface HLA-DR analyzed by flow cytometry. Graph shows values for individual samples and mean [± standard deviation (SD)] and the statistical significance of the differences (Student’s *t*-test). MFI: mean fluorescene intensity

BCR signaling has been demonstrated to upregulate the Ag presentation capacity of normal B cells [33, 34] and we examined the effect of sIgM activation on Ag presentation pathways using beads coated with anti-IgM to mimic particulate Ag [4, 6, 8]. We first performed RNA-seq using 6 sIgM signal responsive CLL samples that were treated with Dynabeads coated with goat polyclonal F(ab’)_2_ anti-human IgM (Go anti-IgM) or control antibody for 6 or 24 h. IPA pathway analysis demonstrated that sIgM stimulation strongly regulated the Ag presentation pathway and it was the most significantly enriched pathway at 6 h (Figure S1; Tables S2 and S3). Analysis of individual genes showed that anti-IgM increased expression of most conventional MHC class II molecules and CIITA (the master regulator of MHC class II expression), as well as calreticulin, calnexin and BiP (HSPA5) (Figure 1A). In contrast, MHC class I molecules were not upregulated following anti-IgM stimulation. Treatment of primary CLL samples with Go anti-IgM beads increased HLA-DR cell surface expression confirming that sIgM stimulation of CLL cells increased MHC class II expression (Figure 1B).

### CLL cells can phagocytose anti-IgM beads

**Figure 2.**
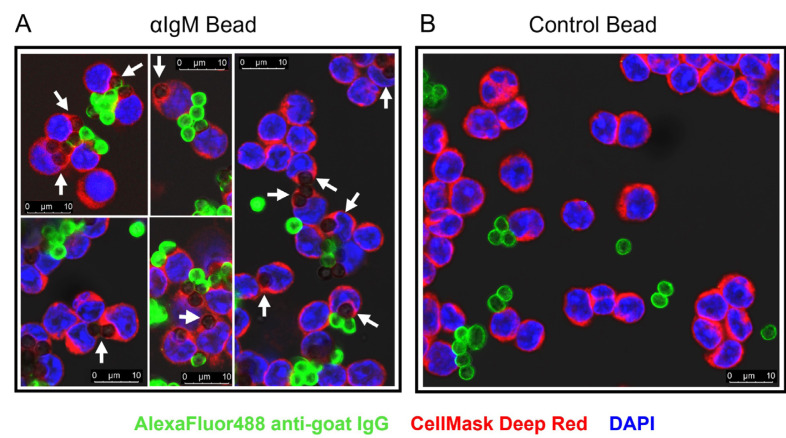
Confocal analysis of phagocytosis of anti-IgM beads by CLL cells. CLL samples were incubated with Go anti-IgM-coated latex beads or control antibody-coated latex beads before analysis of bead internalization by confocal microscopy. A: Five representative merged images for Go anti-IgM-coated latex beads; B: a representative merged image for control antibody-coated latex beads. White arrows indicate internalized Go anti-IgM-coated latex beads (i.e., negative for AlexaFluor488 and engulfed by plasma membrane). Non-internalized anti-IgM or control antibody beads show strong AlexaFluor488 labelling. Note that fewer beads are evident in B as extracellular/unbound beads tended to be lost during preparation of cytospins

We next used confocal microscopy to investigate whether CLL cells were capable of internalizing anti-IgM beads. We adapted an assay that was first described to quantify uptake of anti-IgM beads by normal B cells [8]. CLL cells were incubated with Go anti-IgM beads and then stained with an anti-goat IgG AlexaFluor488 antibody. However, internalized beads remain unlabeled by the AlexaFluor488 antibody as they were protected by the plasma membrane. Experiments were performed using antibodies coupled to 3 μm latex beads to avoid the intense autofluorescence of Dynabeads [35]. Confocal analysis demonstrated that CLL cells internalized anti-IgM beads as we were able to identify unlabeled beads that were engulfed by the plasma membrane (Figure 2A). Approximately 50% of CLL cells had internalized beads and these cells typically contained 1 or 2 beads (Figure S2). In addition to the internalized beads, we were also able to identify beads that were bound to cells but had not been internalized (high AlexaFluor488 fluorescence). Internalization of beads was dependent on anti-IgM as latex beads coated with a control antibody were not internalized (Figure 2B).

**Figure 3.**
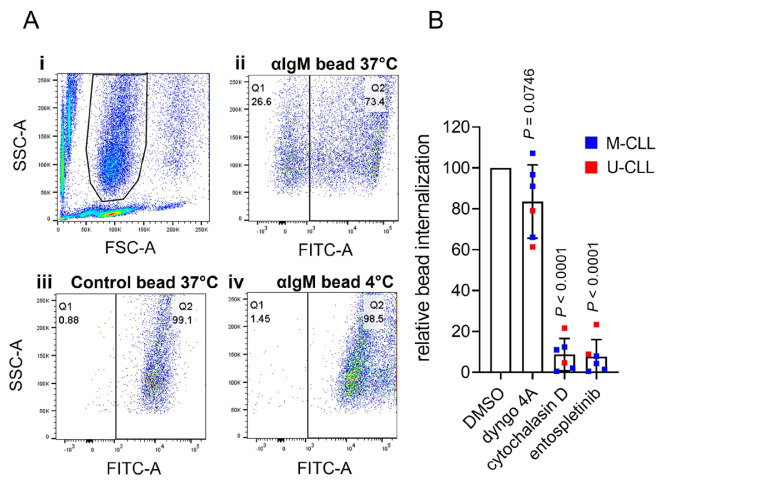
Flow cytometry analysis of phagocytosis of anti-IgM beads by CLL cells. A: (i) Histogram shows gating strategy to identify the beads-and-cells population based on FSC and SSC; (ii)–(iv) fluorescein isothiocyanate (FITC)-SSC dot plots divided (vertical line) into Q1 (cells with internalized beads/low FITC) and Q2 (cells with external beads/high FITC) for (ii) anti-IgM beads at 37°C, (iii) control beads at 37°C or (iv) anti-IgM beads at 4°C. The percentage of events in Q1 and Q2 is shown. Results are shown from a representative sample. Results for an additional 4 samples analyzed are shown in Figure S3; B: CLL samples (*n* = 6) were pre-treated for 1 h with dyngo-4A (10 μmol/L), cytochalasin D (10 μmol/L), entospletinib (1 μmol/L) or DMSO as a control, before analysis of internalization of Go anti-IgM-coated latex beads at 37°C by flow cytometry. Graph shows relative bead internalization with values for DMSO-treated samples set to 100 (with mean ± SD). Results of statistical analysis using one-sample *t*-tests compared to DMSO-treated samples are shown. M-CLL: mutated-CLL; U-CLL: unmutated-CLL

We adapted the assay to analyze internalization using flow cytometry. Following incubation of CLL cells with Go anti-IgM beads, side scatter (SSC)/forward scatter (FSC) analysis revealed three major populations: unbound beads (low FSC), unbound cells (low SSC) and cells-with-beads (Figure 3A). Some of the cells-with-beads population had high AlexaFluor488 fluorescence demonstrating that these cells had cell-surface bound beads that were not internalized (as observed in confocal imaging). However, a proportion of cells had low fluorescence and had therefore internalized beads. This was observed for cells treated with anti-IgM beads at 37°C, but not for control beads at 37°C or for anti-IgM beads at 4°C.

To quantify bead internalization, we calculated the proportion of the cells-with-beads population that had low AlexaFluor488 fluorescence. Although this approach might underestimate the extent of internalization (as it would exclude any cells with, for example, one internalized and one surface-bound bead), it allowed comparison of relative internalization between samples. This demonstrated that although all samples internalized anti-IgM beads, the response varied widely between samples [mean ± SD percentage of cells in Q1 was 23.2 ± 22.1% (*n* = 10; Figure S4)]. Previous studies have shown that phagocytic capacity differs between subsets of normal B cells [8] and our analysis therefore included examples of the two major subsets of CLL, unmutated-CLL (U-CLL) and mutated-CLL (U-CLL), which are derived from pre- and post-germinal centre B cells, respectively [10]. However, the extent of internalization was not statistically significantly different between U- and M-CLL (Figure S4). There was also no statistically significant correlation between internalization and variation in sIgM expression or signaling capacity.

BCR-mediated Ag internalization is classically mediated by clathrin-mediated endocytosis or phagocytosis, depending on the form of Ag [3, 4, 6]. Internalization of anti-IgM beads by CLL cells appeared to be due to phagocytosis as it was effectively inhibited by cytochalasin D, an inhibitor of actin remodeling, whereas the dynamin inhibitor dyngo-4A (which blocks clathrin-mediated endocytosis) had only modest effects (Figure 3B). Anti-IgM bead internalization also required active BCR signalling as it was effectively inhibited by the spleen tyrosine kinase (SYK) inhibitor entospletinib. The modest inhibitory effect of dyngo-4A may reflect a contribution of dynamin to some forms of phagocytosis [36].

### CLL cells can present Ag from anti-IgM beads

Having demonstrated that CLL cells could internalize particulate Ag, we next investigated their ability to present Ag from anti-IgM beads. To quantify MHC class II-restricted presentation, we generated SKW3-T18 cells which express a human TCR specific for a nonamer peptide derived from mouse κ light chains (κLC) when presented on HLA-DRB1*04:01 [29]. Thus, when HLA-DRB1*04:01-expressing CLL cells are exposed to a mouse κLC-containing anti-human IgM F(ab’)_2_ antibody (Ms κ^+^ anti-IgM), CLL cells can internalize and process the mouse antibody and then present the κLC-derived peptide to SKW3-T18 cells. The specificity and sensitivity of the response of SKW3-T18 cells was confirmed by analysis of TCR expression, tetramer staining and response to mouse κLC-derived and control peptides (Figure S5). Analysis of Ag presentation was performed using 2 U-CLL and 2 M-CLL HLA-DRB1*04:01-positive samples and SKW3-T18 cell activation was analyzed by quantification of IL-2 secretion.

**Figure 4.**
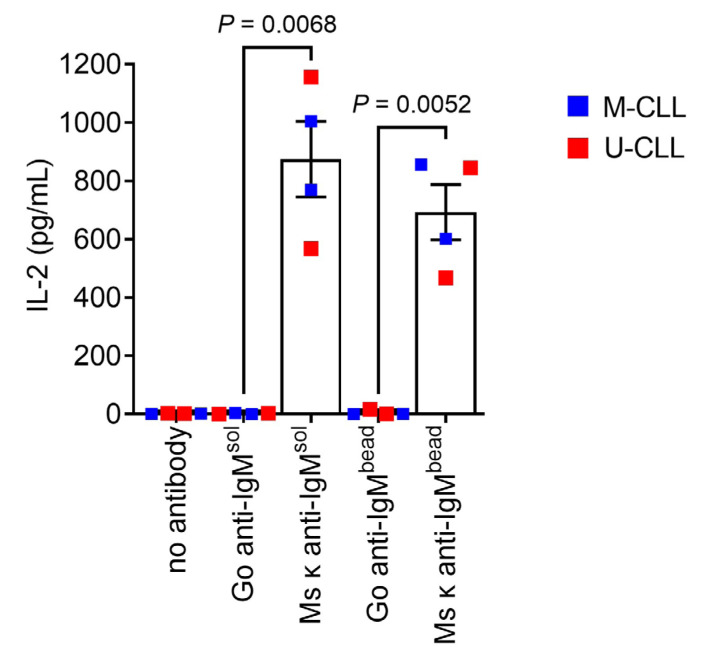
CLL cells can present particulate Ag via sIgM. HLA-DRB1*04:01 positive CLL samples (*n* = 4) were incubated with the indicated bead-bound or soluble antibodies (20 μg/mL), or no antibody as a control, and then co-cultured with SWK3-T18 cells for 24 h. IL-2 secretion was quantified by ELISA. Graph shows results for individual samples (means of duplicates) and mean [± standard error of the mean (SEM)] of results for all samples. The results of statistical comparison are shown (Student’s *t*-tests)

CLL cells incubated with Ms κ^+^ anti-IgM Dynabeads significantly increased IL-2 secretion from SKW3-T18 cells (Figure 4). The magnitude of the response was similar to that obtained with soluble Ms κ^+^ anti-IgM. By contrast, Go anti-IgM (either soluble or bound to Dynabeads), which induces signaling in CLL cells but lacks the mouse κLC-derived epitope, did not induce IL-2 secretion. Therefore, CLL cells can present particulate Ag. Although the number of samples analyzed was small, both U-CLL and M-CLL samples were active in this assay.

**Figure 5.**
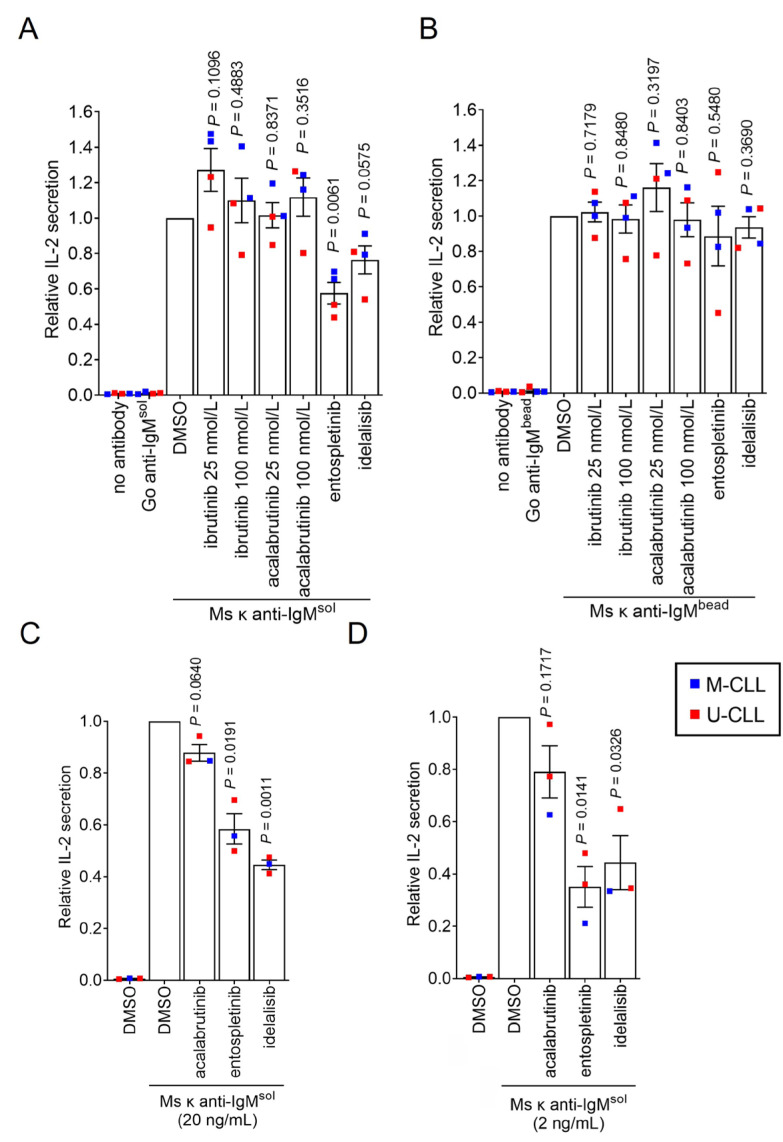
Effect of inhibitors on Ag presentation by CLL cells. CLL samples (*n* = 3 or 4) were pre-treated with ibrutinib (25 and 100 nmol/L), acalabrutinib (25 and 100 nmol/L), entosplentinib (1 μmol/L), idelalisib (1 μmol/L) or DMSO for 1 h before treatment with (A) soluble Ms κ anti-IgM (20 μg/mL), (B) Ms κ anti-IgM-coated beads, (C) soluble Ms κ anti-IgM (20 ng/mL) or (D) soluble Ms κ anti-IgM (2 ng/mL). Cells were then co-cultured with SKW3-T18 cells for 24 h and IL-2 secretion quantified by ELISA. Graphs shows results for individual samples (means of duplicates) and mean (± SEM) for all samples with values for DMSO-treated cells set to 1.0. The results of statistical comparison to DMSO-treated cells are shown (one sample *t*-tests)

We used inhibitors to investigate the role of specific BCR signalling kinases in Ag presentation by CLL cells. The compounds used were the SYK inhibitor entospletinib, the phosphatidylinositol 3-kinase delta (PI3Kδ) inhibitor idelalisib and the BTK inhibitors ibrutinib and acalabrutinib. Compounds were used at concentrations shown to be sufficient for target inhibition in previous studies. IL-2 secretion from SKW3-T18 cells co-cultured with CLL samples treated with soluble Ms κ^+^ anti-IgM was significantly reduced by entospletinib (Figure 5A). IL-2 secretion was also reduced by idelalisib, but the effects of this drug did not reach statistical significance. By contrast, IL-2 secretion was unaffected by ibrutinib or acalabrutinib at either 25 or 100 nmol/L, although concentrations of ibrutinib as low as 10 nmol/L have been shown to be sufficient to occupy the active site of the vast majority of BTK molecules in intact cells [37–40]. Moreover, at 10 nmol/L, ibrutinib and acalabrutinib very effectively suppress BTK-mediated induction of CD69 expression, including following anti-IgM stimulation of CLL cells [37, 41] and ibrutinib and acalabrutinib effectively inhibit anti-IgM-induced downstream phospholipase C gamma 2 (PLCγ2) phosphorylation in B-cell lines and CLL cells (Arthur et al., manuscript in preparation). Thus, the failure of BTKi to inhibit Ag presentation is unlikely to be due to lack of BTK inhibition.

Although entospletinib appeared to reduce IL-2 secretion in one sample (448B), none of the kinase inhibitors had a significant effect on Ag presentation from Ms κ^+^ anti-IgM-coated beads (Figure 5B). Since effects of entospletinib or idelalisib were only observed with soluble anti-IgM, they could not have been mediated by direct effects of these inhibitors on SKW3-T18 cells or cytotoxic effects.

Although we were able to reveal effects of kinase inhibitors on Ag presentation, it was surprising that signalling inhibition only partially reduced (soluble anti-IgM) or had no effect (bead anti-IgM) on Ag presentation by CLL cells to SKW3-T18 cells as signalling has been shown to play a significant role in Ag presentation by normal B cells [4, 5, 42, 43]. One possible reason for this was that the experiments were performed using a relatively large input of anti-IgM, so Ag presentation was hard to inhibit. We therefore repeated the assay using substantially lower concentrations of soluble Ms κ^+^ anti-IgM (20 or 2 ng/mL). This was done using 3 of the HLA DRB1*04:01 positive CLL samples due to limited material available for the fourth. Reducing the amount of antibody used did result in lower levels of IL-2 secretion; mean (± SEM) IL-2 secretion was 131 ± 20 and 37 ± 23 pg/mL for CLL cells treated with 20 or 2 ng/mL soluble Ms κ^+^ anti-IgM, respectively (compared to 875 ± 130 pg/mL for 20 μg/mL). The overall pattern of effects of inhibitors on IL-2 secretion was similar to the initial results although the magnitude of the effects was greater (especially at the lowest concentration of anti-IgM) and in these experiments the inhibitory effects of entospletinib and idelalisib (but not acalabrutinib) were significant (Figure 5C and D).

## Discussion

The BCR plays a critical role in the pathogenesis of CLL and the consequences of BCR signaling have been characterized in detail. By contrast, the ability of CLL cells to utilize the BCR to internalize Ag for presentation to Th cells (which have been implicated as important supporting cells in many studies [12–15]) has not been studied in depth. Our study provides important new insight into the ability of CLL cells to present Ag to Th cells. First, BCR signaling up-regulates the Ag presenting capacity of CLL cells, especially by increasing expression of MHC class II molecules. Second, in addition to being able to present soluble Ag [12], CLL cells are able to phagocytose and efficiently present particulate Ag targeted to sIgM. Although an important caveat of our study is that we have used anti-IgM beads as a surrogate for Ag, this approach has been widely used to model interactions with particulate Ag [4, 6, 8].

We demonstrated that primary CLL cells were capable of internalizing anti-IgM beads. Uptake of anti-IgM beads appeared to be due to phagocytosis as internalization was dependent on actin remodeling and was inhibited by entospletinib, consistent with a requirement for BCR-mediated signaling in the phagocytosis of anti-IgM beads by normal B cells [4]. Although phagocytic activity differs between normal B-cell subsets [44, 45], both M-CLL and U-CLL samples were capable of internalizing (and presenting Ag from) anti-IgM beads. Thus, phagocytosis may be one route by which CLL cells obtain and present particulate Ag. However, it is important to bear in mind that other pathways may operate. For example, normal B cells have also been shown to release lysosome contents into the B-cell synapse following Ag engagement of the BCR, resulting in the extracellular liberation of soluble Ag which can then be internalized or loaded directly into cell surface MHC class II molecules [46, 47]. It is not known whether a similar pathway might also operate for CLL cells.

We used inhibitors to investigate the role of specific BCR-associated kinases in Ag presentation by CLL cells and were able to demonstrate a substantial role for SYK and PI3Kδ, but not BTK, in Ag presentation from soluble anti-IgM. However, the SKW3-T18 assay for Ag presentation appeared to be relatively insensitive to the effects of inhibitors as even the effects of entospletinib were partial, and none of the inhibitors had a significant effect on Ag presentation using anti-IgM beads. The inability of kinase inhibitors to interfere with Ag presentation using anti-IgM beads was particularly surprising as the internalization of anti-IgM beads was almost fully blocked by entospletinib, and the insensitivity of the Ag presentation assay may reflect the use of relatively high amounts of anti-IgM and/or expression of a high affinity TCR by SKW3-T18 cells. Substantially reducing the amount of soluble anti-IgM used in the assay did reduce the degree of T-cell activation and increase the extent of inhibition by entospletinib and idelalisib. However, it was notable that even under these conditions Ag presentation remained relatively unaffected by BTK inhibition. We did not attempt to perform similar experiments for anti-IgM beads as even a low bead to cell ratio (2:1) yielded levels of T-cell activation similar to saturating amounts of soluble anti-IgM and it made little sense to use sub-stoichiometric bead-to-cell ratios. Overall, we favor the explanation that the failure to demonstrate an effect of kinase inhibition on Ag presentation with anti-IgM beads shows that presentation of particulate Ag is highly efficient, rather than the pathway is independent of signalling *per se*.

Overall, our results demonstrate for the first time, that both U-CLL and M-CLL cells are capable of using their BCRs to present particulate Ag. The extent to which CLL cells receive T-cell help *in vivo* remains unclear, and T-cell defects in CLL patients and reduced expression of costimulatory molecules by CLL cells may limit the response [48, 49]. However, T cells derived from CLL patients have been shown to be capable of stimulating proliferation of autologous CLL cells *in vitro* and *in vivo* [12]. Therefore, the ability of CLL cells to present particulate Ag may be important for pathogenesis, especially as some CLL BCRs can recognize Ag/autoantigens (including viruses, bacteria, fungi and apoptotic cell fragments) which may be encountered in a particulate form *in vivo* [19–24] and particulate Ag may engage T-cell help more effectively than soluble Ag. Ag presentation appears to be relatively independent of BTK and may therefore be unaffected in patients treated with ibrutinib or other BTK inhibitors. Future studies of the pathways that mediate Ag internalization and presentation may reveal potential targets for therapies aimed at depriving CLL cells of T-cell help.

## Abbreviations

Ag: antigen
BCR: B-cell receptor
BTK: Bruton’s tyrosine kinase
CLL: chronic lymphocytic leukemia
DMSO: dimethyl sulfoxide
ELISA: enzyme-linked immunosorbent assay
FITC: fluorescein isothiocyanate
FSC: forward scatter
Go anti-IgM: goat polyclonal F(ab’)_2_ anti-human immunoglobulin M
HLA: human leukocyte antigen
IgM: immunoglobulin M
IL-4: interleukin-4
M-CLL: mutated-chronic lymphocytic leukemia
MHC: major histocompatibility complex
κLC: κ light chain
Ms κ^+^ anti-IgM: κ light chain-containing anti-human immunoglobulin M F(ab’)_2_ antibody
sIgM: surface immunoglobulin M
SEM: standard error of the mean
SSC: side scatter
SYK: spleen tyrosine kinase
TCR: T-cell receptor
Th: T helper
U-CLL: unmutated-chronic lymphocytic leukemia

## Supplementary materials

The supplementary material for this article is available at: https://www.explorationpub.com/uploads/Article/file/100270_sup_1.pdf.

## Declarations

### Copyright

© The Author(s) 2022.
