# Supplementary material for: B-cell receptor dependent phagocytosis and presentation of particulate antigen by chronic lymphocytic leukemia cells
Source: Explor Target Antitumor Ther. 2022 Feb 25;3(1):37–49. doi: 10.37349/etat.2022.00070 (PMC7612515; doi:10.37349/etat.2022.00070)
Supplement: Supplementary file 1 [file etat-03-100270-s001.pdf]

B-cell receptor dependent phagocytosis and presentation of particulate antigen by chronic lymphocytic leukemia cells

Annabel R. Minton, Lindsay D. Smith, Dean J. Bryant, Jonathan C. Strefford, Francesco Forconi, Freda K. Stevenson, David A. Tumbarello, Edd James, Geir Åge Løset, Ludvig A. Munthe, Andrew J. Steele, Graham Packham

Supplementary files

Table S1. Details of samples

**Sample**[a]	***IGHV*** **status**[b]	***IGHV*** **usage**[c]	**% CLL cells**[d]	**sIgM signal capacity**[e]	**sIgM MFI**[f]
**87B**	M	IGHV2-5*02 F	95	36	12
**132A**	M	IGHV4-4*07 F	88	18	25
**348C**	M	IGHV3-15*01 F	96	21	35
**348D**	M	IGHV3-15*01 F	98	4	53
**351C**	M	IGHV3-66*01 F, or IGHV3-66*04 F	96	79	34
**448**	U	IGHV3-49*05 F	93	nd	35
**448B**	U	IGHV3-49*05 F	83	73	115
**466**	U	IGHV4-38-2*02 F	81	12	34
**496F**	M	IGHV3-30*03	75	14	32
**511A**	U	IGHV3-72*01 F	96	39	32
**523G**	M	IGHV3-72*01 F	96	13	23
**523H**	M	IGHV3-72*01 F	92	39	45
**575C**	M	IGHV3-15*01 F	96	66	117
**604C**	M	IGHV3-30*03 F, or IGHV3-30*05 F or IGHV3-30*06 F or	85	60	61

		IGHV3-30*13 F or IGHV3-30*18 F or IGHV3-30*19 F			
**621**	M	IGHV1-3*01	95	50	41
**681B**	M	IGHV3-7*02 F, or Homsap IGHV3-7*03 F	98	27	116
**720**	U	IGHV1-69*01 F	92	11	63
**732**	U	IGHV1-46*01 F, or Homsap IGHV1-46*03 F	91	47	85
**774A**	U	IGHV1-69*01 F	97	25	40
**780**	U	IGHV3-21*01 F, or Homsap IGHV3-21*02 F	99	81	65
**888**	U	IGHV3-21*01 F, or Homsap IGHV3-21*02 F	94	65	139
**929**	U	Homsap IGHV1-69*01 F, or Homsap IGHV1-69D*01 F	90	58	134
**1384**	M	IGHV3-33*01 F, or IGHV3-33*06 F	84	21	82
**1427**	U	IGHV1-8*01 F	95	83	1268
**1431**	U	IGHV3-21*01 F, or IGHV3-21*02	97	76	50

Diagnosis of CLL was according to the IWCLL-NCI 2008 criteria and the monoclonal B-cell population in the peripheral blood had a typical $IgM^{+}IgD^{+}$ CLL phenotype in all cases. Where treatment for CLL had taken place, this was at least 6 months prior to sample collection. [a]Where suffix is not shown, this is the first sample obtained from that patient, typically obtained shortly after diagnosis. A, B, C etc. indicate subsequent samples from the same patient. [b]*IGHV* mutation status (M, mutated; U, unmutated) and [c]gene usage. [d]Percentage of CLL cells. [e]Maximal percentage of cells with increased intracellular calcium following treatment with soluble anti-IgM. [f]Mean fluorescence intensity. nd, not determined. Samples 496F, 511A, 348C and 448B were the HLA-DRB1-*04:01 positive samples used for antigen presentation experiments and samples 87B, 132A, 523G, 1384, 1427 and 1431 were used for RNA-seq

Table S2. Top-10 IPA canonical pathways enriched in the anti-IgM-induced transcriptional response at 6 hours

Rank	Ingenuity Canonical Pathway[a]	–log *p*-value	Ratio	z-score[b]	Molecules
**1**	Antigen Presentation Pathway	10.7	0.359	NaN	CALR, CANX, CIITA, HLA-DOA, HLA-DPA1, HLA-DPB1, HLA-DQA1, HLA-DQA2, HLA-DQB1, HLA-DQB2, HLA-DRA, HLA-DRB1, HLA-DRB5, PDIA3
**2**	PD-1, PD-L1 Cancer Immunotherapy	8.47	0.179	-1.604	BCL2L1, CD274, CD80, HLA-DOA, HLA-DPA1, HLA-DPB1, HLA-DQA1, HLA-DQA2, HLA-DQB1, HLA-DQB2, HLA-DRA, HLA-DRB1, HLA-DRB5, IL2RB, PDCD1, STAT5A, TGFB2, TNF, TNFRSF1B
**3**	MSP-RON Signalling in Macrophages	7.73	0.162	-1.886	CIITA, CREB5, HLA-DOA, HLA-DPA1, HLA-DPB1, HLA-DQA1, HLA-DQA2, HLA-DQB1, HLA-DQB2, HLA-DRA, HLA-DRB1, HLA-DRB5, KLK1, KLK2, NFKB1, REL, SOCS3, STAT3, TNF
**4**	Sirtuin Signalling Pathway	7.51	0.106	0.447	DOT1L, ESRRA, GADD45A, GADD45B, GADD45G, LDHA, MAPK12, MAPK6, MYC, MYCN, NAMPT, NFKB1, PCK2, PGAM1, POLR1A, POLR1B, POLR1C, PPIF, REL, RRP9, STAT3, TIMM13, TIMM23, TIMM8A, TIMM9, TNF, TOMM20L, TOMM40, TUBA1B, TUBA1C, VDAC1
**5**	tRNA Charging	7.28	0.282	3.317	AARS1, EARS2, FARSB, GARS1, IARS1, NARS2, SARS1, TARS1, VARS1, WARS1, YARS1
**6**	Th1 and Th2 Activation Pathway	6.91	0.128	NaN	BHLHE41, CD274, CD80, GFI1, HLA-DOA, HLA-DPA1, HLA-DPB1, HLA-DQA1, HLA-DQA2, HLA-DQB1, HLA-DQB2, HLA-DRA, HLA-DRB1, HLA-DRB5, IL2RB, IL6R, NFIL3, NFKB1, SOCS3, STAT3, STAT5A, TNFRSF4
**7**	B cell Development	6.8	0.256	NaN	CD80, HLA-DOA, HLA-DPA1, HLA-DPB1, HLA-DQA1, HLA-DQA2, HLA-DQB1, HLA-DQB2, HLA-DRA, HLA-DRB1, HLA-DRB5
**8**	Purine Nucleotides De Novo Biosynthesis II	6.19	0.545	2.449	ADSL, ATIC, GART, PAICS, PFAS, PPAT
**9**	Th1 Pathway	6.01	0.139	3.051	CD274, CD80, HLA-DOA, HLA-DPA1, HLA-DPB1, HLA-DQA1, HLA-DQA2, HLA-DQB1, HLA-DQB2, HLA-DRA, HLA-DRB1, HLA-DRB5, IL6R, NFIL3, NFKB1, SOCS3, STAT3
**10**	Role of PKR in Interferon Induction and Antiviral Response	5.99	0.132	-0.500	CYCS, DNAJC3, HSP90AA1, HSP90AB1, HSP90B1, HSPA5, HSPA8, HSPA9, MAP2K3, MAPK12, NFKB1, NFKBIE, NLRP6, NPM1, PDGFA, REL, STAT3, TNF

[a]IPA canonical pathway analysis was performed using genes that were significantly up-regulated ($log_2FC > 1.0$; $FDR < 0.05$) at 6 hours in anti-IgM-treated cells. [b]NaN, no activity pattern predicted

Table S3. Top-10 IPA canonical pathways enriched in the anti-IgM-induced transcriptional response at 24 hours

Rank	Ingenuity Canonical Pathway[a]	–log *P*-value	Ratio	z-score[b]	Molecules
**1**	BAG2 Signaling Pathway	11.2	0.25	1.667	CASP3, CDKN1A, HSP90AA1, HSPA2, HSPA5, HSPA8, HSPA9, MYC, NFKB1, PSMB2, PSMB3, PSMB5, PSMB6, PSMB7, PSMC1, PSMC2, PSMC3, PSMD1, PSMD11, PSMD14, REL
**2**	Superpathway of Cholesterol Biosynthesis	10.8	0.448	3.606	ACAT2, CYP51A1, DHCR24, DHCR7, FDFT1, HMGCR, HMGCS1, IDI1, MSMO1, MVD, MVK, NSDHL, SQLE
**3**	Inhibition of ARE-Mediated mRNA Degradation Pathway	8.5	0.155	2.673	EXOSC4, EXOSC5, LTA, MAPK12, MAPK6, PRKAR1B, PRKAR2B, PSMB2, PSMB3, PSMB5, PSMB6, PSMB7, PSMC1, PSMC2, PSMC3, PSMD1, PSMD11, PSMD14, TNF, TNFRSF1B, TNFSF14, TNFSF15, TNFSF4, YWHAE, YWHAG
**4**	Sirtuin Signaling Pathway	8.48	0.12	0	ATP5F1B, ATP5MC1, ATP5PF, GADD45B, GADD45G, LDHA, MAPK12, MAPK6, MYC, MYCN, NAMPT, NDUFA4L2, NDUFAB1, NDUFB3, NFKB1, NQO1, PCK2, PGAM1, PGK1, PPIF, REL, RRP9, SLC25A5, SOD1, SREBF1, TIMM13, TIMM23, TNF, TOMM22, TOMM40, TOMM40L, TP73, TUBA1B, TUBA1C, VDAC1
**5**	Antigen Presentation Pathway	7.81	0.308	NaN	CALR, CANX, CD74, HLA-DPA1, HLA-DQA1, HLA-DQB1, HLA-DRA, HLA-DRB1, HLA-DRB5, PDIA3, PSMB5, PSMB6
**6**	Crosstalk between Dendritic Cells and Natural Killer Cells	7.18	0.187	3.464	ACTB, ACTG1, CAMK2B, CCR7, CD80, CD83, CSF2, FSCN1, HLA-DRA, HLA-DRB1, HLA-DRB5, IL2RB, LTA, NFKB1, REL, TNF, TNFRSF1B
**7**	FAT10 Signaling Pathway	6.79	0.232	NaN	PSMB2, PSMB3, PSMB5, PSMB6, PSMB7, PSMC1, PSMC2, PSMC3, PSMD1, PSMD11, PSMD14, SQSTM1, TNF
**8**	Cholesterol Biosynthesis I	6.75	0.538	2.646	CYP51A1, DHCR24, DHCR7, FDFT1, MSMO1, NSDHL, SQLE
**9**	Cholesterol Biosynthesis II (via 24,25-dihydrolanosterol)	6.75	0.538	2.646	CYP51A1, DHCR24, DHCR7, FDFT1, MSMO1, NSDHL, SQLE
**10**	Cholesterol Biosynthesis III (via Desmosterol)	6.75	0.538	2.646	CYP51A1, DHCR24, DHCR7, FDFT1, MSMO1, NSDHL, SQLE

[a]IPA canonical pathway analysis was performed using genes that were significantly up-regulated ($log_2FC > 1.0$; FDR < 0.05) at 24 hours in anti-IgM-treated cells. [b]NaN, no activity pattern predicted

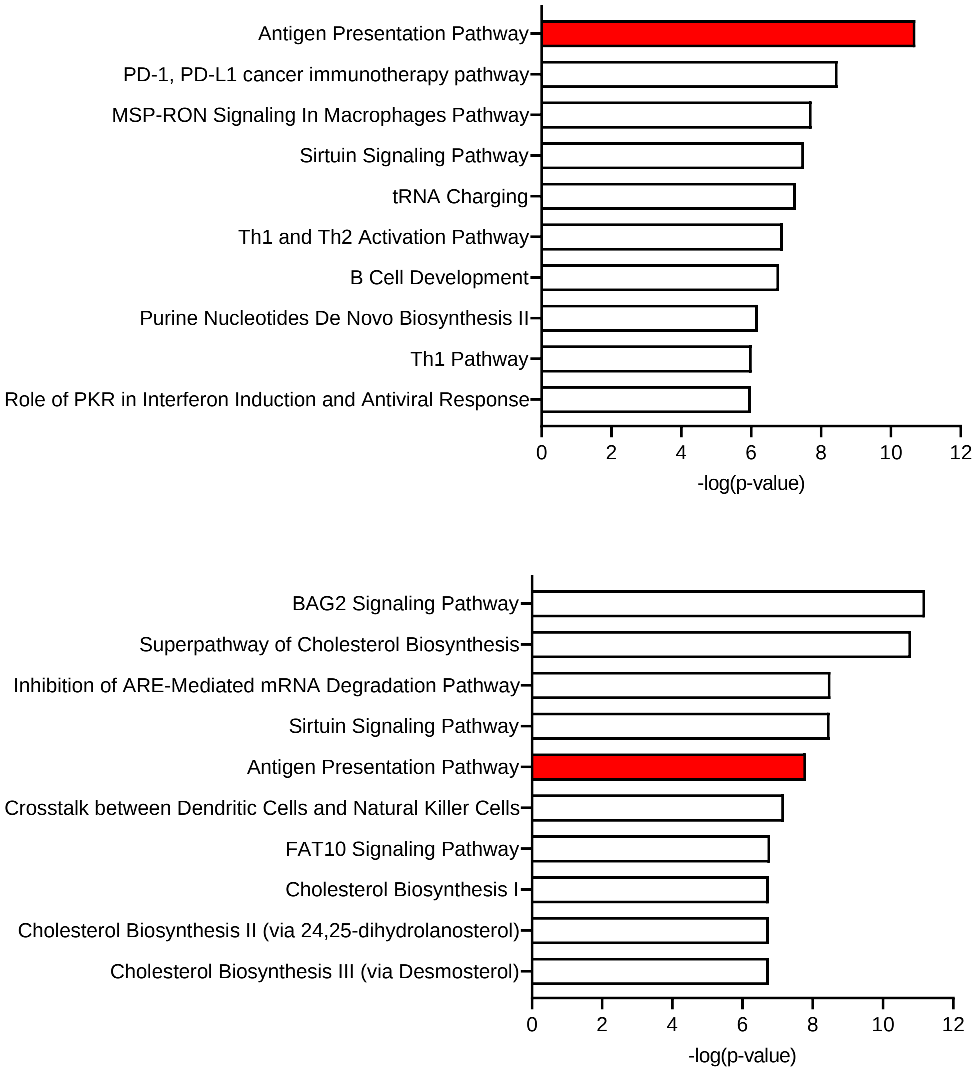

Figure S1. Anti-IgM-regulated pathways. IPA canonical pathway analysis for anti-IgM-induced genes following sIgM stimulation for 6 (upper) or 24 hours (lower). The Ag presentation pathway is highlighted

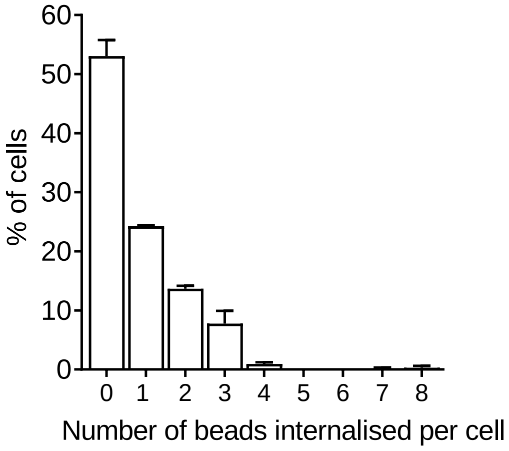

Figure S2. CLL samples were incubated with goat anti-IgM-coated latex beads for 3 hours at 37°C before analysis of bead internalization by confocal microscopy. Graph shows the distribution of the number of internalized beads per cell (mean ± range). Data are from analysis of 2 samples each with 10 representative confocal fields analyzed (mean of 33 ± 15 (SD) cells per field)

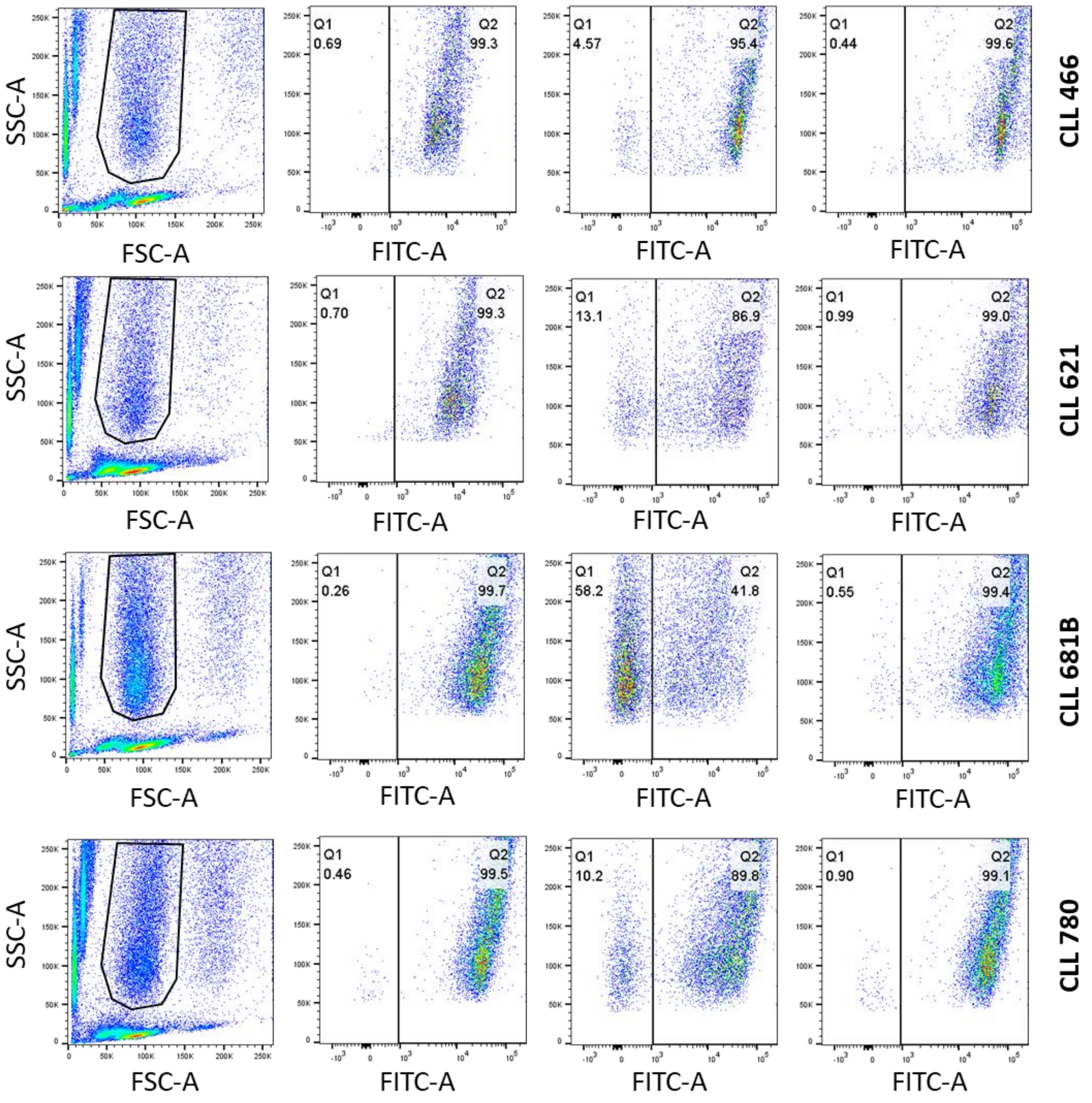

Figure S3. Analysis of anti-IgM bead internalization by flow cytometry. Histogram shows gating strategy to identify the beads-and-cells population based on FSC and SSC. FITC-SSC dot plots divided (vertical line) into Q1 (cells with internalized beads/low FITC) and Q2 (cells with external beads /high FITC) for anti-IgM beads at 37°C, control beads at 37°C or anti-IgM beads at 4°C. The percentage of events in Q1 and Q2 is shown. Results shown are from 4 CLL samples

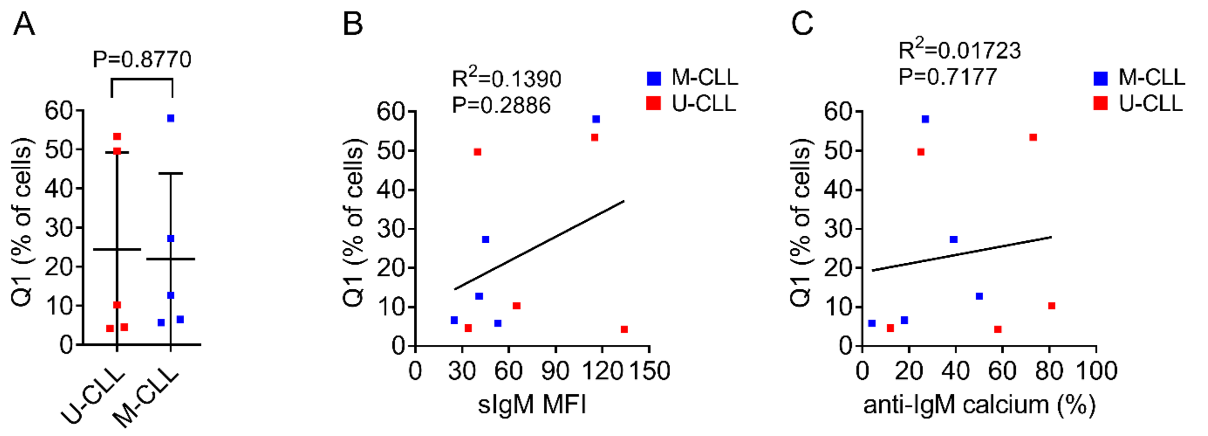

Figure S4. Anti-IgM bead internalization, *IGHV* mutation status and sIgM expression. Graphs show the percentage of cells with internalized beads for all samples analyzed (n = 10) for (A) U-CLL and M-CLL samples (with mean ± SD), (B) sIgM expression and (C) sIgM signalling capacity (anti-IgM-induced calcium mobilization). Results of statistical analysis using (A) Student's t-test or (B,C) Pearson's analysis are shown

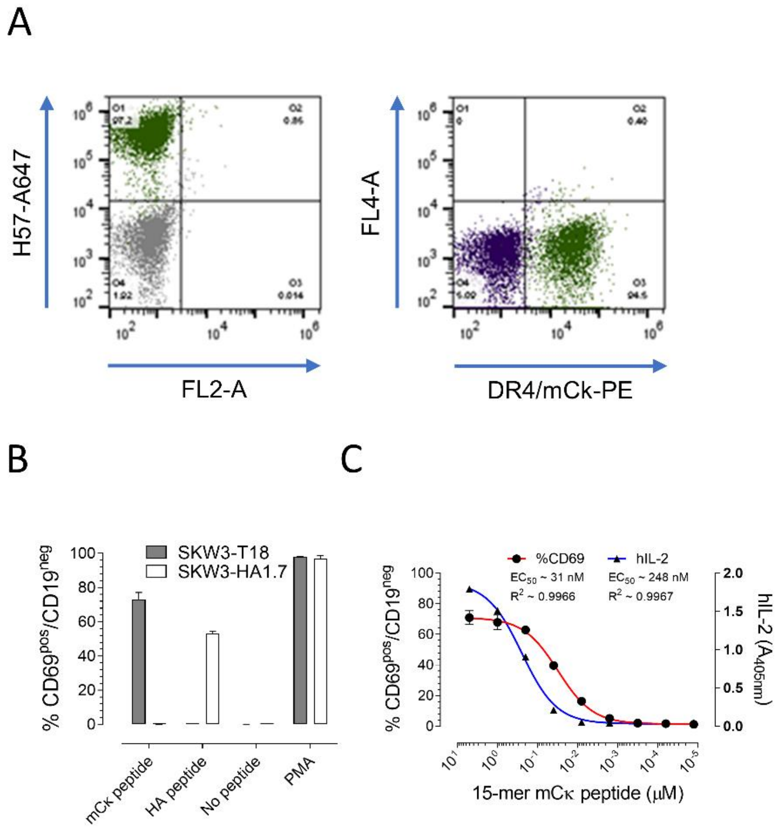

Figure S5. Characterization of SKW3-T18 cells. (A) Flow cytometric analysis of SKW3-T18 cells (green) stained for TCR expression using anti-TCRβ H57-597 antibody (left) or tested for DR4/mCκ-tetramer binding (right). Controls were either parental TCR-negative SKW3 cells (grey) or SKW3 cells expressing an irrelevant TCR (purple); (B) flow cytometric analysis of CD69 expression (percent positive cells) by SKW3-T18 cells or SKW3-HA1.7

cells (expressing the HA1.7 TCR [1]) following incubation with BOLETH cells (ECACC 88052031) loaded with mCk-derived peptide (NVKWKIDGSERQNGV) or HA$^{aa306\text{-}318}$ peptide (PKYVKQNTLKLAT). PMA was used as a positive control for T-cell activation and CD69 expression was quantified on CD19-negative T cells; (C) peptide sensitivity of SKW-T18 cells assessed using serial dilution of mCk-derived peptide loaded on BOLETH cells. Graph shows representative results for cell surface CD69 expression (flow cytometry) and IL-2 secretion (ELISA)

Supplementary Reference

1. Hennecke J, Carfi A, Wiley DC. Structure of a covalently stabilized complex of a human alphabeta T-cell receptor, influenza HA peptide and MHC class II molecule, HLA-DR1. EMBO J. 2000;19:5611–24.
